# Efficient utilization of red mud waste via stepwise leaching to obtain α-hematite and mesoporous γ-alumina

**DOI:** 10.1038/s41598-023-35753-w

**Published:** 2023-05-26

**Authors:** Zahra Karimi, Ahmad Rahbar-Kelishami

**Affiliations:** School of Chemical, Petroleum and Gas Engineering, Iran University of Science and Technology (IUST), Narmak, Tehran, Iran

**Keywords:** Chemical engineering, Civil engineering

## Abstract

Utilizing the red mud sustainably is now a challenging issue. Red mud due to its wide production, presence of some radioactive elements, high alkalinity, and salinity, has a dramatic potential to contaminate soil and groundwater. Notwithstanding its drawbacks, Red mud consists several elements, including Ca, Al, Ti, Si, and Fe, in various mineral forms. In this study, stepwise leaching was applied as a proper method to separate and purify the main valuable elements using available and affordable HCl. The pre-leaching step under optimized conditions using HCl (0.2 M) at room temperature for 2 h removed 89% of the calcium content from red mud. To selectively remove the solid silica, the residue was treated with concentrated HCl (3.0 M, L/S of 20 mL/g) at 95 °C, resulting in the dissolution of iron and aluminum content with up to 90% efficiency. After precipitation of the Fe^3+^ and Al^3+^, they were characterized using FT-IR, BET, EDS, XRD, SEM and TEM monographs, confirming the formation of nanosized hematite (α-Fe_2_O_3_) and mesoporous gamma alumina (γ-Al_2_O_3_). Consequently, inexpensive red mud was converted into highly valuable nano-sized metal oxides using simple, sustainable techniques and cheap reagents. Moreover, this technique generates the lowest amounts of waste during the leaching process and all reagents can be recycled for further uses, making this method a sustainable utilization.

## Introduction

Red mud, also referred to as bauxite residue, is the largest process waste produced during alumina production from bauxite ore by the Bayer method. Approximately 1–2 tons of red mud are produced for every ton of alumina produced^1^. Global red mud manufacturing is anticipated to be 200 million tons annually. Red mud accumulation has surpassed 4.6 billion tons, and the world’s red mud stockpile is steadily expanding^2,3^. Red mud is considered a significant environmental threat due to its high alkalinity (pH 10–13), high salinity, and the existence of certain radioactive elements, including scandium, gallium, uranium, and thorium. These factors increase the likelihood of soil and groundwater contamination, which can harm both the local population and the ecosystem as a whole^4^.

In the years ahead, there will likely be a global increase in the demand for aluminum, which will boost the production of red mud. This will impede the alumina industry's ability to grow sustainably^5^. Furthermore, red mud production is predicted to rise in the future as the grade of bauxite ore declines as a result of early extraction of the highest-quality bauxite sources, resulting in a higher ratio of red mud production to bauxite^6^. The most pressing issue facing the global aluminum industry is how to properly dispose of red mud. A wide range of disposal methods are available, including dry stacking, seawater discharge, lagooning, and the like. These ways are not only harmful, but they also end up wasting precious resources^7^.

Red mud has been the subject of extensive research in a variety of areas to find a sustainable method for the usage of red mud, including its application in soil improvement, metal recovery and steel making, catalytic reactions, low-cost adsorbents for pollutant removal, the production of tiles, ceramics, and bricks, the production of pigments and paints, and slag additives^1^.

Metal recovery from red mud has been considered noteworthy since this substance is a potential alternative source of Fe_2_O_3_, Al_2_O_3_, CaO, and SiO_2_, and some valuable metal oxides, such as rare-earth oxides^8^. Studies are predominantly based on hydrometallurgical (leaching, solvent extraction and precipitation) and pyro-metallurgical/mechanical operations (magnetic separation and sintering, reductive smelting, roasting), or combinations thereof. While hydrometallurgical processes are still promising owing to their potential for selective metals recovery, the high efficiency and desirable environmental aspects, pyro-metallurgical processes are still problematic due to their high energy consumption, production of toxic gases, and worthless residues^9–11^. Recent studies have shown that hydrometallurgical treatment using various acids brings about different results for the extraction of different elements. In this regard, metals recovery from red mud by selective leaching has been reported mostly by sulfuric acid^11–17^, hydrochloric acid^9,18–20^, nitric acid^11,21^, phosphoric acid^11,22,23^, and oxalic acid^24–27^. Pepper et al.^11^ examined the selectivity of HCl, HNO_3_, H_2_SO_4_, and H_3_PO_4_ in red mud extraction experiments. The acquired results revealed that nitric acid has an acceptable efficiency in extracting aluminum and silica from red mud. In contrast, phosphoric and hydrochloric acids represented the higher recovery efficiencies for Fe and Ti. Iron extraction as high as 47% was obtained using refluxing H_2_SO_4_ (8 N) at 100 °C with a solid-to-liquid ratio of 0.05 g/mL during 24 h^28^. The removals of 97.46% iron and 64.40% aluminum were reported by Uzun and Gülfen^29^ using 6 M sulphuric acid from red mud calcined at 600 °C. In another study^30^, the maximum SiO_2_ recovery of 80% from red mud has been accomplished based on a two-step leaching process with dilute HCl and concentrated H_2_SO_4_. The results displayed that the maximum Fe_2_O_3_ and Al_2_O_3_ recovery of 95.4% and 66.7% could be obtained respectively, upon leaching with HCl (3 M) with a solid–liquid ratio of 1:16 g/mL, refluxing at 90 °C for 90 min. Wang et al.^31^ leached red mud with HCl (3 M) at 100 °C for 2.0 h. The leaching process resulted in high content of iron and also low content of other elements.

Most of the applicable methods are based on the recovery of only one metal oxide that constitutes the largest percentage of red mud, while multi-element recovery from red mud would have more potential for industrial application and provide more benefits. In this research, we focused on a stepwise method for the recovery of alumina, hematite, calcium, and silica from red mud. This method includes a pre-leaching step using HCl (0.2 M) to exclude calcium contents, a leaching step using HCl (3 M) to exclude silica content, treatment with ammonia followed by NaOH to extract iron content, and finally treatment with HCl (3.0 M) to give alumina. The developed method led to the formation of γ-Al_2_O_3_ and α-Fe_2_O_3_, both of which are valuable minerals. γ-Al_2_O_3_ is widely applied as adsorbent, catalyst, catalyst support, and coating. In addition, hematite is a valuable commercial product in the cosmetic and pigment industries^32,33^.

The two-stage extraction process developed in the present work provides several advantages and innovations, including: (1) It operates at a low temperature, under atmospheric pressure, resulting in safe and cost-effective conditions. (2) It generates considerably fewer environmental hazards in comparison to the conventional method, which is due to the low concentrations of the leachate and precipitation. (3) HCl, as a cheap acid, is manufactured on a large scale as a by-product of many chemical industries and can be neutralized as a safe waste. (4) The products, including γ-Al_2_O_3_ and α-Fe_2_O_3_, have a noticeably higher economic value than the low-cost red mud and reagents. (5) The four main elements of red mud that constitute the highest percentages are separated step by step, generating the lowest level of waste and no contaminated waste, is produced during this process.

## Materials and methods

### Materials

#### Chemical reagents

Hydrochloric acid (HCl, analytical grade), ammonia (NH_4_OH, analytical grade), sodium hydroxide pellets (NaOH, analytical grade), and ferrous sulfate (FeSO_4_, analytical grade) were purchased from Merck Company and used with no further purification.

#### Red mud

The red mud used in this study was obtained as a solid residue from the Iran Alumina Company, located in Jajarm, northeast of Iran. The chemical composition of the as-received red mud was determined using XRF, as shown in Table 1. The as-received red mud contains majorly iron oxide, calcium oxide, aluminum oxide, silicon, and sodium oxide. Moreover, there are small amounts of titanium, and magnesium oxides and much less zirconium.

**Table 1:** The Chemical composition of as received red mud determined by XRF.

Composition	Al_2_O_3_	SiO_2_	Fe_2_O_3_	TiO_2_	CaO	MgO	Na_2_O	K_2_O	SO_3_	P_2_O_5_	Cl	Zr
Mass %	19.21	16.79	23.55	5.38	21.17	1.45	9.90	0.59	0.69	0.15	0.98	0.14

**Figure 1:**
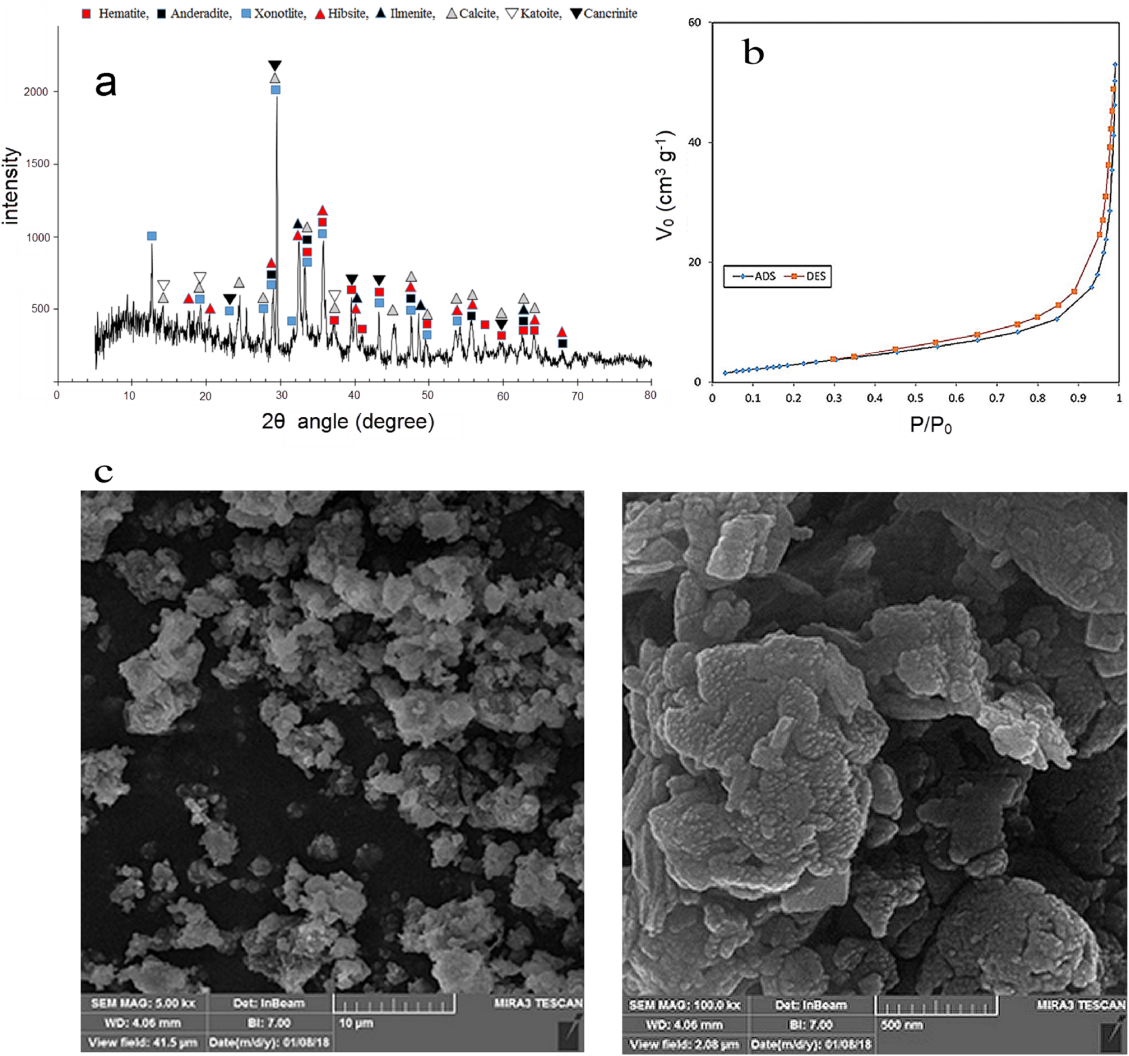
(**a**) XRD pattern of red mud, (**b**) N_2_ sorptometry of red mud, (**c**) FE-SEM images of red mud.

The mineralogical phases of Jajarm Red Mud were examined using X-ray powder diffractometry. Results of the XRD pattern in Fig. 1a show the existence of hematite, andradite, katoite, hibschite, ilmenite, and xonotlite in the initial red mud. Figure 1b shows the nitrogen sorptometry curve of the as received red mud. As can be seen, the red mud has a small mesopore volume. From matching the nitrogen sorptometry data with the BET isotherm, the total pore volume and specific surface area values were 0.08 cm^3^/g and 12.3 m^2^/g, respectively, indicating that the red mud is relatively non-porous. As shown in Fig. 1c, the red mud has a dense and nonporous microstructure. According to the particle size distribution of the red mud of the Iran Alumina Company, all the particles have a size below 24 µm, and half of them have a size less than 2.94 µm^1^.

### Methods

#### Experimental procedure

The study was carried out in a 500 mL glass reactor connected to a 40 cm glass Graham condenser and placed on a heater with a magnetic stirring system. The stirring speed was maintained constant during experiments to ensure that the particles remained suspended. Moreover, the magnetic stirrer was equipped with a temperature probe. Water bath was used in terms of homogenization of reaction temperature.

The stirring speed was maintained constant at 500 rpm during experiments to ensure that the particles remained suspended. The following Eq. (1) was applied to calculate the efficiency of dissolution:

1 $$ {\text{DE}} = \left[ {\left( {{\text{W}}_{{\text{i}}} - {\text{W}}_{{\text{r}}} } \right)/{\text{W}}_{{\text{i}}} } \right]*{1}00 $$

where DE is dissolution efficiency and W_i_ and W_r_ are mass of initial red mud and mass of solid residue, respectively.

**Figure 2:**
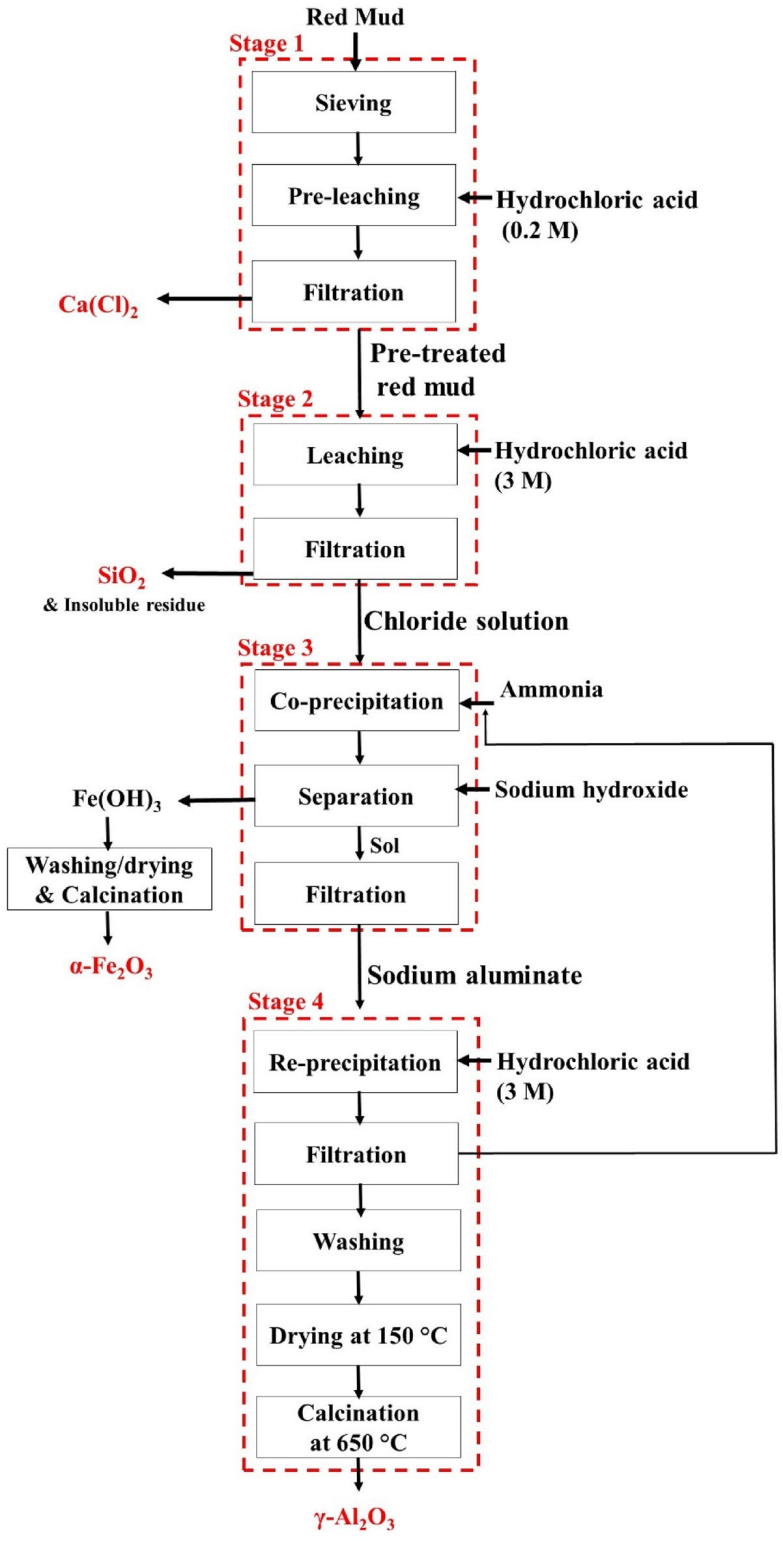
The flowchart of alumina and iron oxide extraction from red mud.

Sampling was accomplished using the coning and quartering method^34^. Having enough dried samples in hand, they were passed through a standard sieve with a hole diameter of less than 710 μm using dry method. The experimental procedure includes 3 parts: Pre-leaching, main leaching and co-precipitation. Figure 2 illustrates the flowchart of the proposed process. It must be noted that the metal contents of samples were determined by XRF after each leaching step.

##### Pre-leaching

In this step, separately a specified amount of hydrochloric acid of known concentration (0.2 M, 100 mL) was poured into the flask. At ambient temperature, a certain amount of completely dried red mud (5.0 g) was added and stirred well for 2 h under refluxing conditions. Upon completion of the pre-leaching experiment, the resulting suspension was filtered under vacuum condition to separate the solid residue from the leachate. The acquired solid residue was well washed several times with distilled water, then dried at 110 °C for 12 h and finally weighed. The HCl-washing led to the removal of 89% calcium content and from red mud due to the occurrence of the following reaction between acid and calcium oxide Eq. (2):

2 $$ {\text{CaO }} + {\text{ 2HCl }} \to {\text{ CaCl}}_{{2}} \left( {{\text{aq}}.} \right) \, + {\text{ H}}_{{2}} {\text{O}} $$

##### Main leaching

In a three-neck round-bottom glass reactor, HCl (3 M, L/S ratio of 20 mL/g) was added to the obtained solid from pre leaching step and stirred well for 2 h at 95 °C under refluxing conditions. This HCl treatment dissolves all metal cations leaving a greenish yellow silica gel residue that can be filtered. The filtrate contained a high concentration of Fe and Al.

##### Co-precipitation

The obtained filtrate was titrated with ammonia (25%) at room temperature. An unstable gel was formed at pH 4. The titration was continued until a brownish, stable gel with a large amounts of Al(OH)_3_ and Fe(OH)_3_, and a minor amount of Ti(OH)_4_ and Ca(OH)_2_ was formed at pH 9.

3 $$ {\text{MCly }} + {\text{ yNH}}_{{4}} {\text{OH }} \to {\text{ M}}\left( {{\text{OH}}} \right){\text{y}} \downarrow \, + {\text{ yNH}}_{{4}} {\text{Cl}}\,\, \left( {{\text{M }} = {\text{ metal cation}}} \right) $$

To separate Al from Fe and other metals, NaOH (3 M) was added to the mixture of metal hydroxides until its pH reached 14. At this point, the Al(OH)_3_ is converted to a brownish, soluble NaAlO_2_, while other hydroxides remain insoluble. Subsequently, the mixture was filtered.

4 $$ {\text{Al}}\left( {{\text{OH}}} \right)_{{3}} + {\text{ NaOH }} \to {\text{NaAlO}}_{{2}} \left( {{\text{aq}}.} \right) \, + {\text{ 2H}}_{{2}} {\text{O}} $$

The solid comprising iron hydroxide in high percentage and trace amounts of Ca(OH)_2_ and Ti(OH)_4_ was thoroughly washed with freshly distilled water to remove the Cl ions, before being dried and calcined at 500 °C to produce Fe_2_O_3_. Subsequently, the clear, colorless filtrate containing sodium aluminate, was then titrated with HCl (3.0 M) at 25 °C until the solution pH reached 9, resulting in the formation of the Al(OH)_3_ gel, according to the Eq. (5).

5 $$ {\text{NaAlO}}_{{2}} + {\text{ HCl }} + {\text{ H}}_{{2}} {\text{O }} \to {\text{ Al}}\left( {{\text{OH}}} \right)_{{3}} \downarrow \, + {\text{ NaCl }}\left( {{\text{aq}}.} \right) $$

To remove chloride ions, the filtered gel of Al(OH)_3_ was well washed with deionized water until the output water doesn’t show the presence of chlorine. To test this, after every cycle of washing, one drop of AgNO_3_ was added to the output water; we stopped washing when silver chloride precipitate did not form. Then it was dried in an oven at 105 °C, and finally calcined with heating rate of 10 °C/min in air up to 650 °C. After reaching this temperature, the sample left to cool down naturally in the closed furnace to obtain alumina according to the Eq. (6).

6 $$ {\text{2Al}}\left( {{\text{OH}}} \right)_{{3}} \to {\text{ Al}}_{{2}} {\text{O}}_{{3}} + {\text{ 3H}}_{{2}} {\text{O}} $$

#### Characterization techniques

The analysis of red mud’s composition was carried out by X-ray fluorescence (XRF) spectrometer (Philips, Netherland) based on ISO/IEC 17025:2005 standard. Philips Expert System X-ray diffractometer (XRD) was employed for the mineralogical study with CuKa radiation and Ni-filter at 40 kV and 30 mA, at 2Ɵ range of 5°–80° with a scanning rate of 2°/min, an anti-scatter and receiving slit of 1° and 0.01 mm, respectively. Dynamic light scattering (DLS) technique was employed to find the distribution of particle size of red mud, using a scatteroscope I device (DLS, Nanotrac Wave from Microtrac Company). To study the morphology of samples, FESEM (SIGMA VP-500, ZEISS, Germany) was applied at an accelerating voltage of 15 kV. The elemental mapping and energy-dispersive X-ray spectroscopy (EDX) spectra were accomplished using Energy Dispersive X-ray Spectroscopy probe (Oxford Instruments, England). Transmission electron microscopy (TEM, Tecnai F30, Philips, Netherland) and scanning electron microscopy (SEM, TESCAN MIRA3 Microscope, Netherland) were utilized to investigate the morphology. The specific surface area and porosity of red mud, γ-Al_2_O_3_ and α-Fe_2_O_3_ were determined by nitrogen sorptometry analysis at 77 K performed with the BELSORP MINI II, Japan. Before any nitrogen sorptometry test, the samples were degassed at 180 °C for 3 h.

## Results and discussion

### The necessity of two-stage extraction

As shown in Table 2, a literature survey reveals a wide variety of chemical compositions reported for red mud, whereas the highest percentages are attributed to iron, aluminum, silicon, and calcium The proportion of iron varies from 4.52 to 50.06% depending on the texture of the bauxite utilized^35^. The adoption of a standard extraction procedure is complicated by changes in the phase compositions and crystallography of various red mud s. Stepwise leaching seems to be a good choice to overcome this challenge that was first accomplished in this research to separate the Al and Fe oxides from the texture of red mud. The main aim of leaching was to remove the silica and calcium step by step. Selective leaching is not applicable via a one-step process, because it causes to the dissolution of other valuable metal ions together with the calcium ions. In this study, it was discovered that about 89% of the calcium content can be removed via mild acidic leaching by dissolving Ca^2+^ ions in the aqueous phase. Subsequently, more than 95% of the silica content was removed as a solid precipitation in the aqueous solution of metal ions. Finally, the iron and alumina were separated by successive precipitation.

**Table 2:** Major compositions of various red mud sources worldwide (in wt%).

Entry	Country	The Contents (%)						References
		Al_2_O_3_	SiO_2_	Fe_2_O_3_	TiO_2_	CaO	Na_2_O	
1	Iran	19.21	16.79	23.55	5.38	21.17	9.90	Present study
2	Hungary	14.8	13.5	42.1	5.2	6.1	8.2	^36^
3	Russia	11.20	8.72	50.00	4.05	10.76	7.09	^37^
4	Germany	16.20	5.40	44.80	12.33	5.22	4.00	^38^
5	Brazil	15.10	15.60	45.60	4.29	1.16	7.50	^38^
6	Greece	16.26	6.97	42.34	4.27	11.64	3.83	^20^
7	USA	18.4	8.5	35.5	18.4	7.7	6.1	^39^
8	India	16.58	8.32	36.26	17.10	1.43	6.00	^40^
9	Turkey	20.24	15.27	39.84	4.15	1.8	9.43	^39^
10	Jamaica	13.2	3	49.4	7.3	9.4	4	^36^
11	Australia	23.53	14.88	36.48	6.84	1.83	9.41	^41^
12	China, Guizhou	20.73	17.19	20.74	5.29	15.85	6.39	^42^
13	China, Guangxi	24.03	26.80	6.96	3.42	15.58	7.15	^41^
14	South Korea	18.34	6.04	42.52	7.05	9.13	7.07	^14^
15	Ghana	51.07	2.75	7.15	1.77	1.07	2.84	^43^
16	Italy, Sardinia	17.91	9.58	30.45	8.61	7.77	12.06	^44^
17	Bosnia and Herzegovina	16.9	21.9	37.9	-	10.0	7.2	^45^

### The effect of various factors on the pre-leaching step

Four components, including Fe_2_O_3_, CaO, Al_2_O_3_, and SiO_2_, constitute the major part of red mud. One of the most challenging steps was to choose a method to separate calcium from the mixture. In this regard, a comprehensive study was conducted on the impacts of pre-leaching time and temperature, HCl concentration, and liquid to solid (L/S) ration through a One-Factor-At-a Time (OFAT) approach^46^. As Table 3 depicts, various ranges of the mentioned factors were examined to optimize the calcium separation from red mud.

**Table 3:** The ranges of factors to optimize the pre-leaching step.

Factor	Range
Leaching time	0.5–3 h
Leaching temperature	25–85 °C
HCl concentration	0.1–0.5 M
L/S ratio	10–50 mL/g

#### The effect of temperature

**Figure 3:**
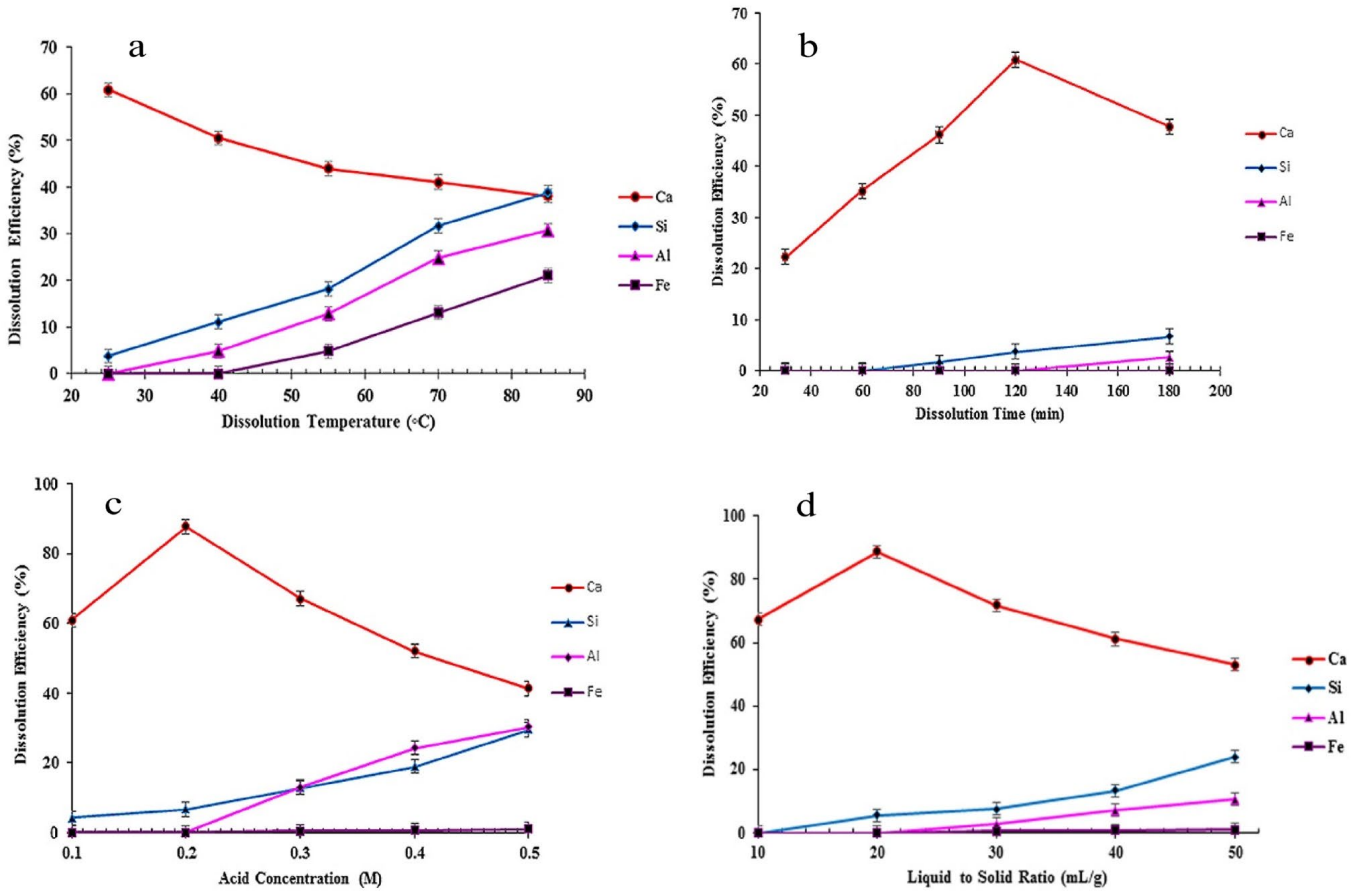
The effect of various factors on the dissolution of Ca, Fe, Al, and Si in HCl (100 mL): (**a**) the temperature (2 h, HCl 0.1 M); (**b**) the time (at 25 °C, HCl 0.1 M); (**c**) the HCl concentration (at 25 °C, 2 h); (**d**) the L/S ratio (at 25 °C, HCl 0.1 M, 2 h).

It was truly predicted that concentrated HCl would dissolve more cations. Therefore, this study was started with the addition of 100 mL of the very diluted HCl (0.1 M) to 5.0 g of red mud to be refluxed at various temperatures for 2 h (Fig. 3a). It was found that at 25 °C, more than 60% of calcium content can be removed, while less than 5% of Al and iron leach into the solution. The higher the temperature, the more leaching of Fe and Al, and the less leaching of calcium. The amounts of leached Fe, Al, and Ca oxides at 85 °C are approximately 20, 30, and 40%, respectively. As a result, 25 °C was found to be the best temperature for the most efficient calcium separation.

#### The effect of time

To evaluate the efficiency of leaching time on the calcium separation, 100 mL of the very diluted HCl (0.1 M) was added to red mud (5.0 g) under refluxing condition at 25 °C (Fig. 3b). By increasing the leaching time up to 2 h, the separation efficiency of calcium increases. Thereafter it decreases. The reason for efficiency drop is that Al and Fe would react with HCl, leading to competition between metal cations. Therefore, a leaching time of 2 h was selected for the remaining examinations.

#### The effect of HCl concentration

This study was performed by varying the HCl concentration while the temperature was adjusted at 25 °C for 2 h. Regarding the results depicted in Fig. 3c, by increasing the acid concentration from 0.1 to 0.5 M, the efficiency of the calcium separation initially increases, then drops considerably. As mentioned above, a higher concentration of HCl can dissolve other species, leading to a competition between cations to attract chloride ions, which results in the lower release of calcium into the solution. Therefore, the proper concentration of HCl was 0.2 M to examine further studies.

#### The effect of liquid to solid ratio

After optimizing the treatment conditions, including the addition of HCl (0.2 M) at 25 °C for 2 h, the optimized ratio of acid volume to the grams of red mud must be obtained. Figure 3d shows that by increasing the L/S ratio up to 50 mL per gram of red mud, the efficiency of calcium oxide separation rises, then falls precipitously. This descent arises from the increase in the numbers of proton and chloride ions, resulting in an increase in the reaction rate, a decrease in selectivity, and an increase in competition between cations. Hence, the best L/S ratio is 20 mL of HCl (0.2 M) per gram of solid. As a result, leaching 5.0 g of red mud in 100 mL of HCl (0.2 M) at 25 °C for 2 h removes 89% of the calcium content, while releasing negligible amounts of alumina and iron oxides.

### The effect of various factors on the main leaching step

Another OFAT study was also accomplished to optimize the leaching process. In this step, all the remaining contents must be dissolved in HCl, but Si. To find the best conditions for dissolution of both alumina and iron oxide contents in their maximum amounts, similar factors mentioned above with different ranges were examined (Table 4).

**Table 4:** The ranges of factors to optimize the pre-leaching step.

Factor	Range
Leaching time	0.5–3 h
Leaching temperature	25–95 °C
HCl concentration	1–7 M
L/S ratio	5–40 mL/g

#### The effect of time

**Figure 4:**
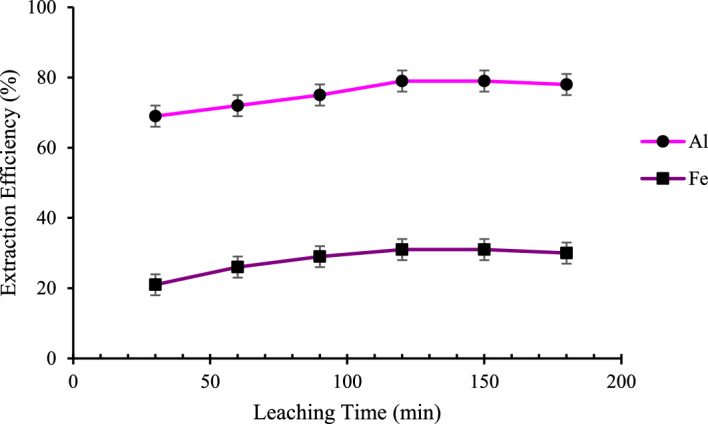
The effect of time on the Fe and Al leaching; HCl (3.0 M, L/S of 20 mL/g), at 25 °C.

To evaluate the efficiency of leaching time on the dissolution of Al^3+^ and Fe^3+^, HCl (3.0 M) with a L/S ratio of 20 mL/g was added to the mixture at 25 °C (Fig. 4). By increasing the leaching time up to 2 h, the dissolution efficiency of both metals increases. Thereafter it decreases. Therefore, a leaching time of 2 h was selected for the remaining examinations.

#### The effect of HCl concentration

**Figure 5:**
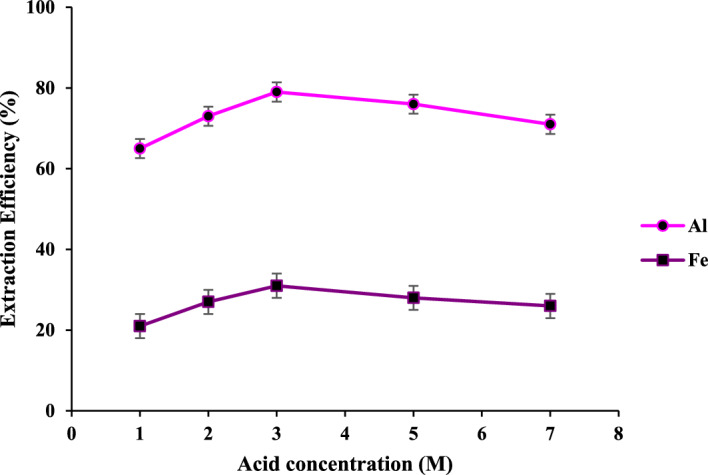
The effect of HCl concentrations (20 mL) on the Fe and Al leaching; L/S of 20 mL/g at 25 °C for 2 h.

Literature survey shows that high amounts of HCl can dissolve all the remaining cations but silica^47,48^. In this study, HCl (20 mL) was added to 1.0 g of the obtained, dried solid and refluxed at 25 °C for 2 h (Fig. 5). By increasing the acid concentration from 1.0 to 3.0 M, the dissolution efficiency for Al^3+^ and Fe^3+^ increases up to 75 and 30%, respectively. While efficiency slightly drops in higher acid concentrations, this is likely due to three factors^49,50^: (a) Higher acid concentrations contain less ionized species^51^, which has an adverse effect on cations leaching. (b) At higher HCl concentration, a burst leaching causes an instant increase in cation concentrations around the solid, reducing the leaching rate due to the blockage of proton penetration. (c) Although lower amounts of competitive metal oxides are present in the current solid, they can still react with concentrated HCl to be leached into the solution, resulting in a lower release of Al^3+^ and Fe^3+^.

#### The effect of liquid to solid ratio

**Figure 6:**
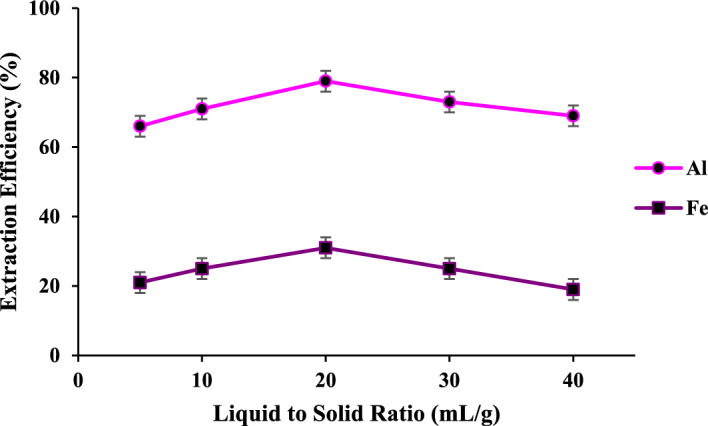
The effect of L/S ratio on the Fe and Al leaching; HCl (3.0 M), at 25 °C for 2 h.

Figure 6 shows the optimization of the L/S ratio for leaching the Al and Fe cations in HCl (3.0 M). By increasing the L/S ratio up to 20 mL per gram of solid, the leaching efficiency of iron and Al species rises up to 30 and 75%, respectively. The higher L/S ratio, the lower the leaching efficiency. This descent arises due to the facts mentioned above. Moreover, an increase in the numbers of proton and chloride ions in comparison with the present metal species in the solid, resulting in an increase in the reaction rate, a decrease in selectivity, and an increase in competition between the desired and unwanted cations. Hence, the best L/S ratio is 20 mL of HCl (3.0 M) per gram of solid.

#### The effect of temperature

**Figure 7:**
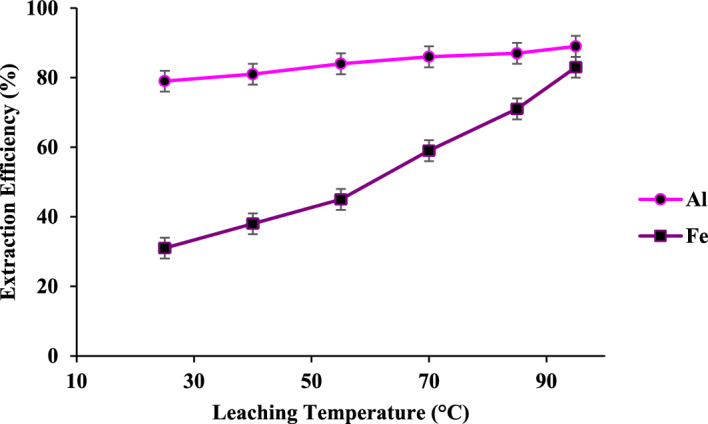
The effect temperature on the Fe and Al leaching, HCl (3.0 M, L/S of 20 mL/g) for 2 h.

To increase the efficiency of leaching of Fe along with the Al, the leaching temperature was optimized as shown in Fig. 7. By increasing the temperature from 25 to 95 °C, the leaching efficiency of Fe increases meaningfully, while it has a small effect on the dissolution of Al. This shows that the dissolution rate of iron is controlled by the interface chemical reaction^47,52,53^. In general, leaching the solid in concentrated HCl (3.0 M, L/S of 20 mL/g) at 95 °C under refluxing conditions for 2 h dissolves Al and Fe cations with up to 90% efficiency.

**Figure 8:**
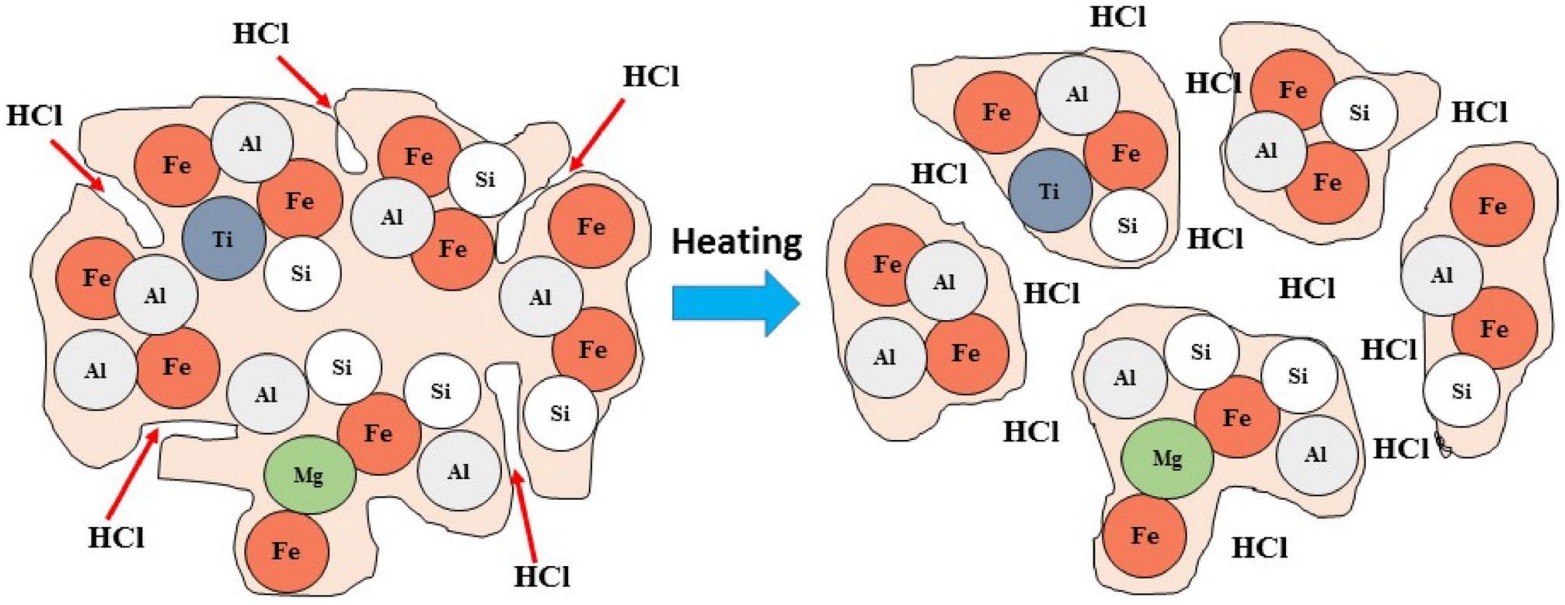
Dissolution of pretreated red mud at temperatures higher than 50 °C under acidic conditions.

It must be noted that at higher temperatures, the dissolved HCl molecules in water are released as gaseous HCl molecules, penetrating the pores of solids due to the increasing pressure. This leads to the acidic decomposition of the solid into the finer particles. Consequently, the increased contact surface of particles with acid is increased, reaching to the untreated core (Fig. 8)^54^. Moreover, it is known that at high temperatures, the acid molecules are broken down into energetic, reactive radical species, entering the mineral lattice^55^ that can improve the dissolution of Al^3+^ and Fe^3+^ during the acid-leaching process.

### Characterization

#### Fourier transform infrared spectroscopy

**Figure 9:**
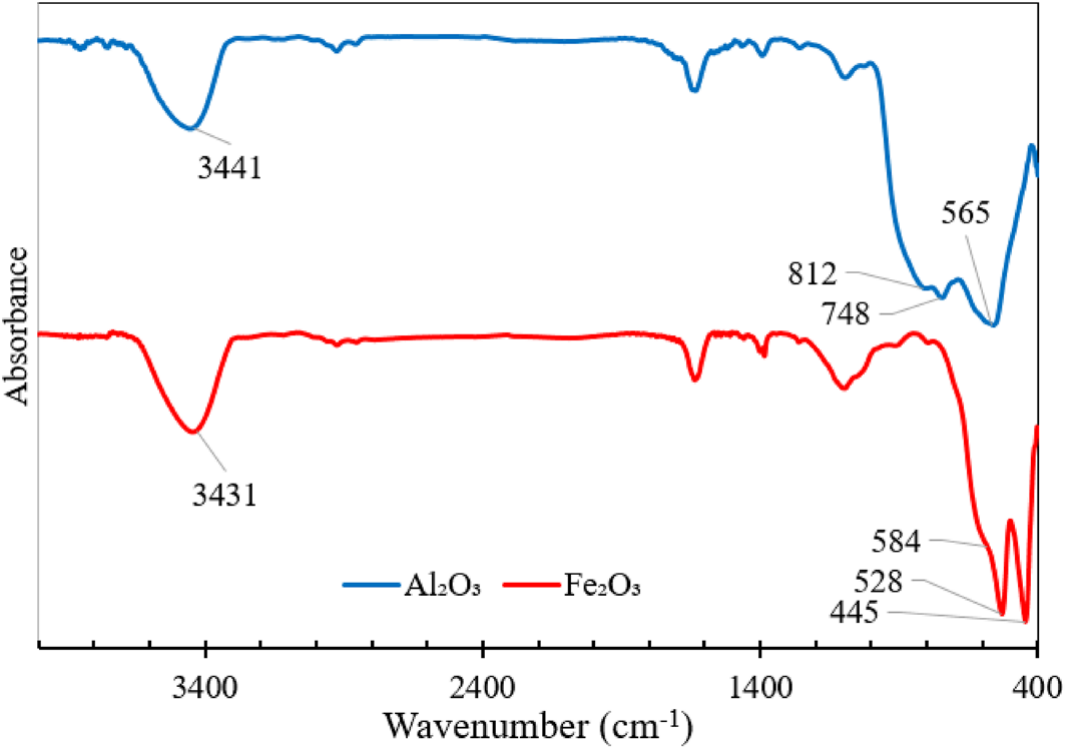
FT-IR spectra of as-synthesized Al_2_O_3_ and Fe_2_O_3_.

Figure 9 shows the FT-IR spectra of as-synthesized alumina and iron oxide. A broad band around 3441 and 3431/cm is assigned to the O–H stretch vibrations on the surfaces of Fe_2_O_3_ and Al_2_O_3_, respectively. The absorbance bands at 584, 528, and 445/cm correspond to the stretching and bending vibrations of Fe–O in hematite (α-Fe_2_O_3_)^56^. The observed bands at 811 and 565/cm are attributed to the stretching vibrations of Al–O–Al, confirming the presence of γ-Al_2_O_3_^57^.

#### XRD patterns

**Figure 10:**
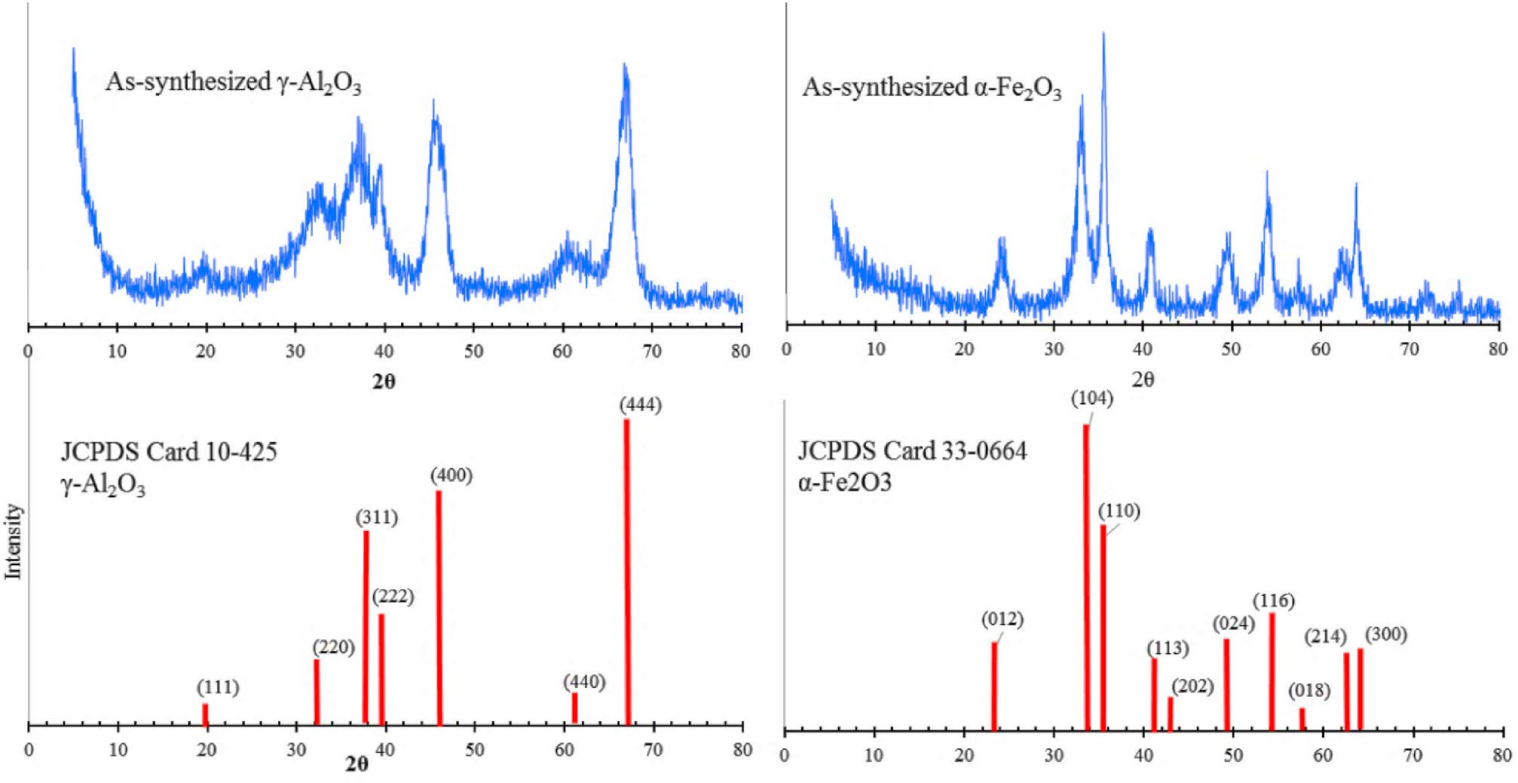
XRD patterns of as-synthesized γ-Al_2_O_3_ and α-Fe_2_O_3_ and their standard patterns.

Figure 10 depicts the XRD patterns of the as-synthesized alumina and iron oxide with widened diffraction lines caused by the extremely small particle size. XRD pattern is the best way to confirm the structure and phase of chemical products. Based on the standard pattern of γ-Al_2_O_3_ (JCPDS No. 10-425), the peaks appeared at 2θ = 19.5°, 32.5°, 37.6°, 39.6°, 52.5°, 45.9°, 61.1°, and 67.1°, which can be indexed to the (111), (220), (311), (222), (400), (440), and (444) lattice planes, respectively, confirming the formation of γ-Al_2_O_3_^58,59^. Furthermore, the XRD pattern of as-synthesized iron oxide is observed in the standard patter (JCPDS No. 33-0664) of hematite, indicating it is α-Fe_2_O_3_^59^.

#### Morphology and characteristics

**Figure 11:**
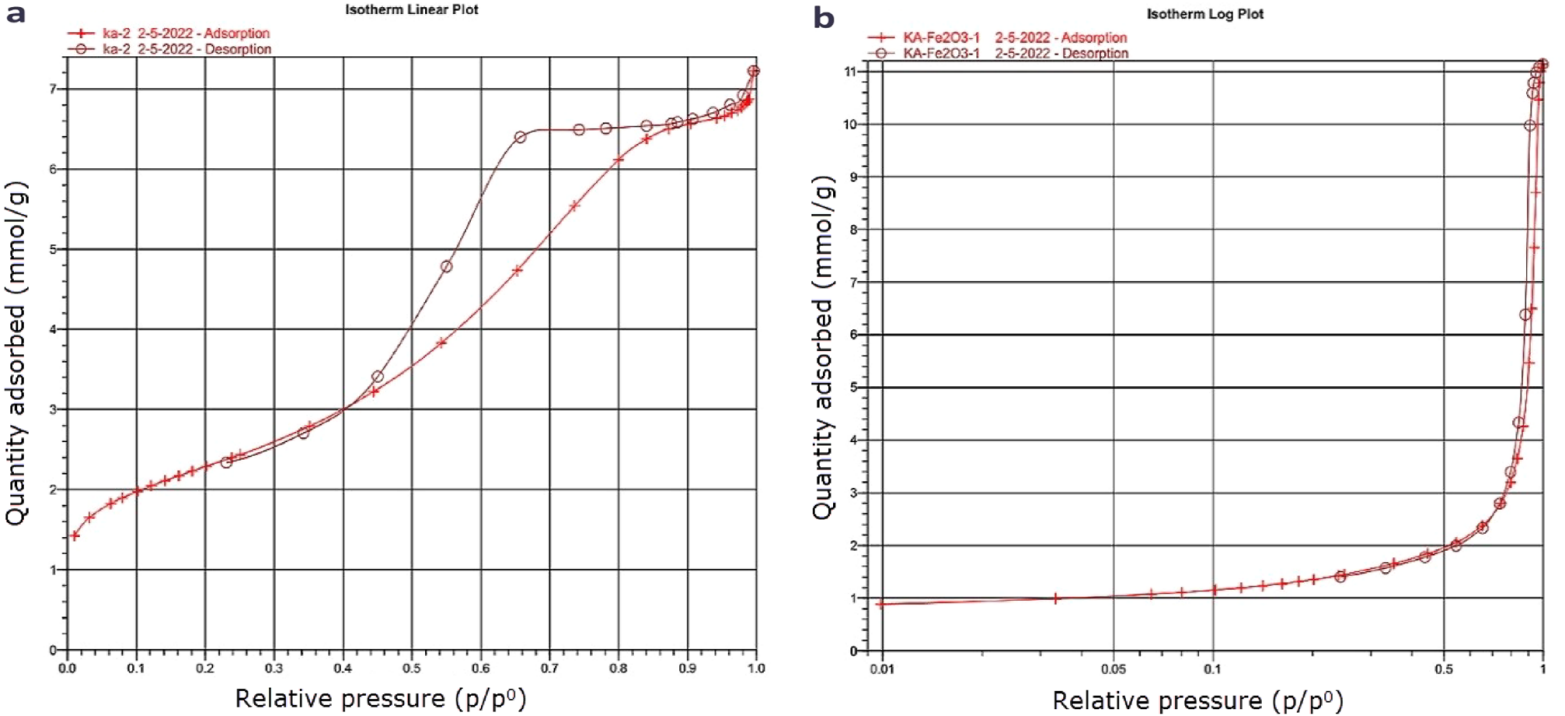
N_2_ adsorption–desorption isotherms of (**a**) as-synthesized γ-Al_2_O_3_, (**b**) and α-Fe_2_O_3_.

The N_2_ adsorption–desorption isotherm (Fig. 11) of the as-synthesized γ-Al_2_O_3_ is a typical irreversible IV-type isotherm with an IUPAC defined hysteresis loop of H1. Therefore, this γ-Al_2_O_3_ is a mesoporous material with a BET surface area of 183.4 ± 0.5 m^2^/g, and its pore volume and pore diameters were obtained by the BJH technique, as summarized in Table 5. The obtained results are comparable with the reported analyses^60^. This confirms that the synthesized alumina is a quasi-ordered γ-Al_2_O_3_, resulting in the broad peaks in its XRD as mentioned before^61,62^. BJH adsorption pore distribution was calculated to confirm the mesoporous characteristics of alumina, showing pore width range from 1.7 to 300 nm. The isotherm of α-Fe_2_O_3_ confirms that this synthesized oxide is non-porous with a BET surface area of 108.6 ± 0.5 m^2^/g.

**Table 5:** The BET surface area and pore characteristics obtained from N_2_ adsorption–desorption.

Sample	S_BET_ (m^2^/g)	Pore volume (cm^3^/g)	Average particle size (nm)	Average pore size (nm)
γ-Al_2_O_3_	183.4	0.24	32.7	5.2
α-Fe_2_O_3_	108.6	0.003	55.2	-

**Figure 12:**
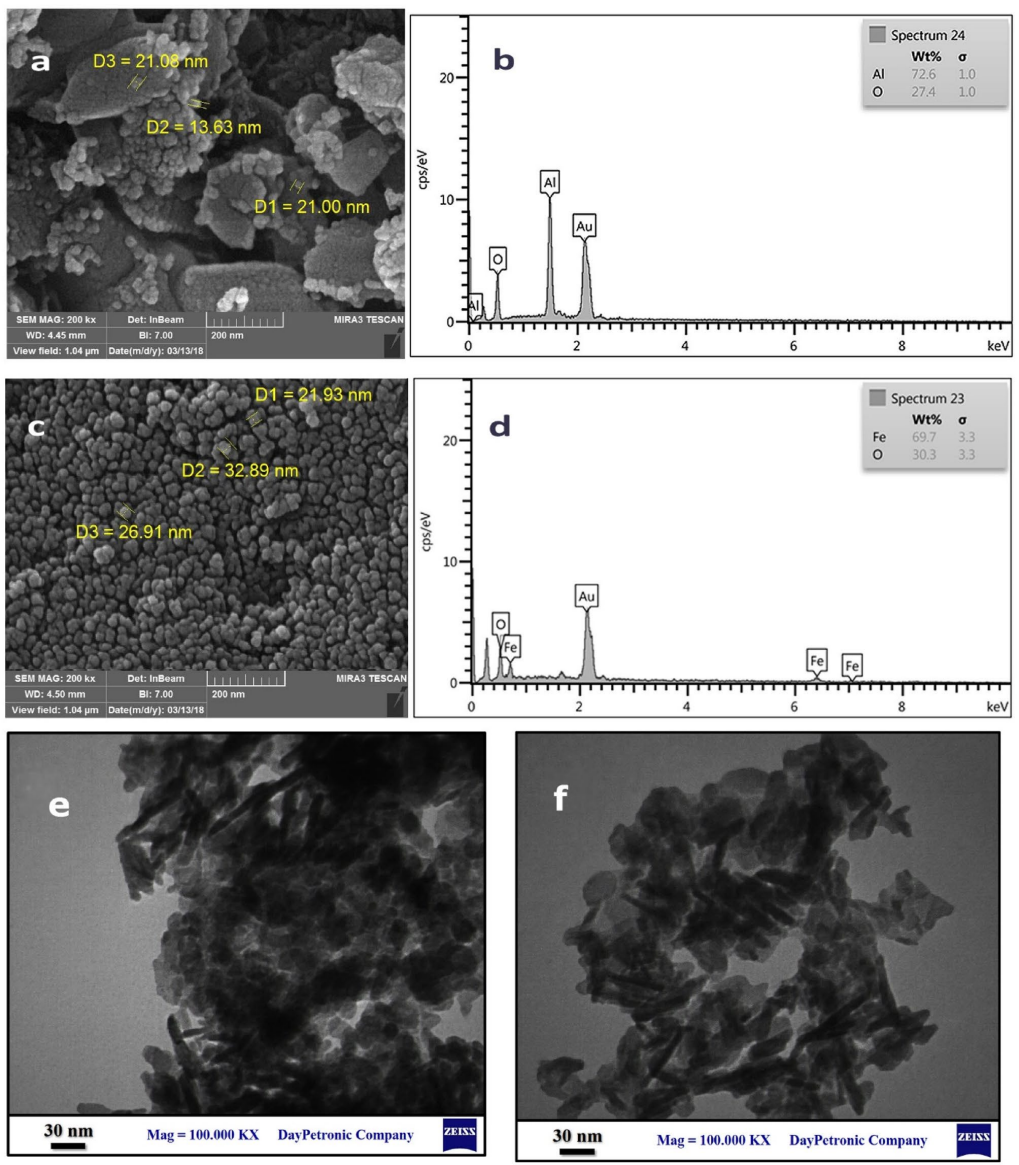
FESEM image and EDS analysis of γ-Al_2_O_3_ (**a**, **b**) and α-Fe_2_O_3_ (**c**, **d**); TEM of γ-Al_2_O_3_ (**e**, **f**).

The morphological and elemental characteristics of γ-Al_2_O_3_ and α-Fe_2_O_3_ were provided by FESEM and EDS analyses. Figure 12a and c shows the FESEM micrographs of the as-synthesized γ-Al_2_O_3_ and α-Fe_2_O_3_ and their respective EDS analyses. Figure 12a depicts agglomerated particles of alumina with different sizes which are composed of very fine particles smaller than 30 nm. The EDS elemental analyses show the extra high purity of the as-synthesized metal oxides (Fig. 12b and d).

Moreover, the TEM image of alumina (Fig. 12e and f) illustrates the agglomerated grain-like particles with an average size of 30 nm. The reported TEM images of gamma alumina confirm that the as-synthesized alumina by our successive leaching method is a gamma mesoporous alumina, confirming its mesoporous structure obtained by BET^63,64^. It is worth noting to say that this studies’method is simple and uses inexpensive reagents to obtain expensive nanosized γ-Al_2_O_3_ while the reported methods for the preparation of such compounds rely on the use of special costly techniques and reagents^65,66^.

## Conclusion

Iron, aluminum, calcium, and silica constitute about 80% of the red mud obtained from the Iran Alumina Company located in Jajarm. This study was able to approach a simple, cheap, and stepwise leaching method for the separation of the main composition of the red mud. Initially, diluted HCl was used to pre-leach the CaO content. Then, concentrated HCl dissolved the iron and aluminum content to separate the solid silica. The characterization of the formed iron oxide and alumina revealed that this simple, successive method leads to the formation of nano-sized α-Fe_2_O_3_ and mesoporous γ-Al_2_O_3_ with particle sizes lower than 30 nm. Hematite is a highly demanded form of iron oxide in the cosmetic and pigment industries. Moreover, gamma alumina is an expensive kind of alumina that is widely utilized in the production of ceramics, hybrid and phosphorescent pigments, and industrial catalysts. It is noteworthy to mention that the common methods for the preparation of gamma alumina need costly reagents and complicated equipment, whereas this method converts the discounted red mud, obtained from alumina industry waste, into the invaluable γ-Al_2_O_3_ using affordable HCl.

## Data Availability

All data generated or analyzed data for the experimental part of this study are included in this published article. The data that support the findings of this study are available from the corresponding author, [Ahmad Rahbar-Kelishami], upon reasonable request. Moreover, all other data that support the plots within this paper and other findings of this study are available from the corresponding author upon reasonable request.
